# Detecting Emotional Contagion in Massive Social Networks

**DOI:** 10.1371/journal.pone.0090315

**Published:** 2014-03-12

**Authors:** Lorenzo Coviello, Yunkyu Sohn, Adam D. I. Kramer, Cameron Marlow, Massimo Franceschetti, Nicholas A. Christakis, James H. Fowler

**Affiliations:** 1 Electrical and Computer Engineering Department, University of California San Diego, San Diego, California, United States of America; 2 Political Science Department, University of California San Diego, San Diego, California, United States of America; 3 Facebook Inc., Menlo Park, California, United States of America; 4 Department of Sociology, Yale University, New Haven, Connecticut, United States of America; 5 Department of Medicine, Yale University, New Haven, Connecticut, United States of America; 6 Medical Genetics Division, School of Medicine, University of California San Diego, San Diego, California, United States of America; University of Namur, Belgium

## Abstract

Happiness and other emotions spread between people in direct contact, but it is unclear whether massive online social networks also contribute to this spread. Here, we elaborate a novel method for measuring the contagion of emotional expression. With data from millions of Facebook users, we show that rainfall directly influences the emotional content of their status messages, and it also affects the status messages of friends in other cities who are not experiencing rainfall. For every one person affected directly, rainfall alters the emotional expression of about one to two other people, suggesting that online social networks may magnify the intensity of global emotional synchrony.

## Introduction

Happiness and other emotions have recently been an important focus of attention in a wide range of disciplines, including psychology, economics, and neuroscience [1], [2], [3], [4]. Some of this work suggests that emotional states can be transferred directly from one individual to another via mimicry and the copying of emotionally-relevant bodily actions like facial expressions [5]. Experiments have demonstrated that people can “catch” emotional states they observe in others over time frames ranging from seconds to months [6], [7], and the possibility of emotional contagion between strangers, even those in ephemeral contact, has been documented by the effects of “service with a smile” on customer satisfaction and tipping [8].

Longitudinal data from face-to-face social networks has established that emotions as diverse as happiness [9], loneliness [10], and depression [11] are correlated between socially-connected individuals, and related work suggests that these correlations also exist online [4], [12], [13], [14], [15]. However, it is difficult to ascertain whether correlations in observational studies result from influencing the emotions of social contacts (contagion) or from choosing social contacts with similar emotions (homophily) [16].

This problem has been addressed by using experimental methods to estimate network effects [17], [18], [19], [20], [21], [22], but these methods are either limited in scale and external validity, or they require very close collaboration with private companies, which means there are limited opportunities to conduct such experiments. Moreover, even when companies are willing to conduct a large-scale experiment, they may have other goals that constrain its design. For example, they may wish to provide a uniform online experience to all users, which reduces their willingness to create experimental treatment groups of sufficient size to take advantage of their massive scale.

Here, we propose an alternative method for detecting emotional contagion in massive social networks that is based on instrumental variables regression, a technique pioneered in economics [23]. In an experiment we would directly control each user's emotional expression to see what impact it has on their friends' emotional expression. However, since this is infeasible in our massive-scale setting, we identify a source of variation that directly affects the users' emotional expression (this variable is called an “instrument”). For this instrument, we use rainfall. Importantly, rainfall is unlikely to be causally affected by human emotional states, so if we find a relationship it suggests that rainfall influences emotional expression and not vice versa. We then measure whether or not the changes induced by the instrument predict changes in the friends' emotional expression. Instead of changing the user's emotion directly with an experimental treatment, we let rainfall do the work for us by measuring how much the rain-induced change in a user's expression predicts changes in the user's friends' expression.

More formally, suppose we can represent one person's emotional expression mathematically as follows:
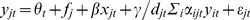
(1)This model assumes that emotional expression *y_jt_* by person *j* at time *t* is an additive linear function of other factors measured in the same time period including a time-specific factor *θ_t_* (perhaps it is a holiday), an individual-specific factor *f_j_* (some people are always happier than others), the effect *β* of an exogenous factor *x_jt_* (like rainfall); the effect *γ* of an endogenous factor *y_it_* (the emotional expression of each friend *i* at time *t*), which is moderated by the strength of relationship *a_ijt_* between each friend *i* and person *j* at time *t* (for simplicity, we assume this is binary—a relationship either exists or it does not) and by the degree *d_jt_* of person *j* (a person with more friends is assumed to be less influenced by each); and an error term *ε_jt_*. The key variable allowing us to estimate contagion in emotional expression is *γ*, and our inclusion of the individual-fixed effect *f_j_* means that we are controlling for all possible characteristics of the person, which further reduces the likelihood that correlation in emotions is driven by choice of social connections (homophily).

Although this model seems straightforward to estimate, it is not. The reciprocal influence of a user on her friend and vice versa makes it difficult to interpret a simple association in their emotional states. Moreover, in large populations, it would be computationally expensive to apply the model to longitudinal content generated by millions of users with billions of friends over thousands of days.

We address the problem of computational cost by aggregating individuals into groups. In the supporting information (Text S1), we show that when a subpopulation of individuals experience the same exogenous factor (such as rainfall affecting individuals who are in the same city), equation (1) is equivalent to
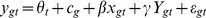
(2)where for time *t*, *y_gt_* is the average emotion of all people in subpopulation (city) *g*; *θ_t_* and *c_g_* are time and subpopulation fixed effects; *x_gt_* is the average exogenous factor (rainfall) for people in subpopulation *g*; *Y_gt_* is a weighted average emotional expression of friends of people in subpopulation *g*; and *ε_gt_* is an error term (see Text S1 for derivation).

Notice that we can use equation (2) to estimate the social contagion effect *γ* that appears in equation (1). However, *y_gt_* and *Y_gt_* are still endogenous, so prior to estimation we use an instrument *X_gt_*, the aggregated rainfall of the friends of the people in subpopulation *g*, to predict exogenous variation in the friends' emotional expression *Y_gt_*:
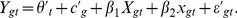
(3)


Consistent with standard recommendations regarding instrumental variable regression [23], we include in the “first stage” equation (3) all other exogenous explanatory variables in the “second stage” equation (2). Thus, we are estimating the effect of rainfall on average emotion while controlling for time and city fixed effects and for rainfall in all other cities. This mitigates problems that may arise from autocorrelation in weather over time and between nearby cities. We then use predicted values from equation (3) to substitute for the value of *Y_gt_* in equation (2) to estimate the social contagion variable *γ*. This instrumental variables approach effectively addresses the problem of endogeneity (in our case, the fact that two friends influence one another) [23].

One worry in a model like this is that friends' rainfall is correlated, so the instrument might actually just be a proxy for the direct effect of rainfall on a person's emotional expression (a violation of the “exclusion restriction” [23]). Therefore, to break any possible correlation between rainfall *x_gt_* in city *g* and the rainfall *X_gt_* of their friends, we only consider how emotional expression is transmitted on days when it is not directly raining on city *g* (that is, we only include observations for which *x_gt_* = 0, see Text S1). Then, in a separate model, we consider only days when it is raining in city *g*.

Another worry is that there is an “ecological fallacy” in this model since we are using city-level measures to estimate individual-level effects. In Text S1 we mathematically formalize the relationship between the individual and aggregate level models to show there is no problem in our case, but here we explain in words. The ecological fallacy occurs when there are opposing effects of individual-level and aggregate-level variation. For example, Robinson showed that U.S. states with more immigrants had higher literacy rates (perhaps because literate state populations were more tolerant of immigration), even though immigrants were less likely to be literate (perhaps because they had not yet learned English) [24]. However, a key factor that reduces the likelihood this is a problem in our model is that people in a city usually all experience the same weather on the same day, so city-level variation is a good predictor of individual-level variation (if you were in New York on a given day you probably experienced the same weather as everybody else in New York that day). Compare this to the Robinson example, where state-level immigration rates are a very poor predictor of individual-level immigrant status, which allows for the possibility of opposite correlations with literacy at the aggregate and individual level.

We apply our method to data collected for a set of 1180 days on Facebook from January 2009 to March 2012. The study was approved by and carried out under the guidelines of the Institutional Review Board at the University of California, San Diego, which waived the need for participant consent. To protect participant confidentiality, researchers did not personally view any names of users or words posted by users, and all analysis of identified data took place in the same secure location on servers where Facebook currently keeps users' data.

Users of Facebook interact with each other in many ways, mostly textual. To measure emotional expression, we use “status updates” (also called “posts”) which are undirected text-based messages that a user's social contacts (Facebook friends) may view on their own News Feed. Relying on the Linguistic Inquiry Word Count (LIWC), a widely used and validated word classification system [25],[26], we determine whether a post uses words that express positive or negative emotions. Although this is not the only way to measure sentiment [27], this method has previously been used to measure the emotional content of online messages [28]. We then use two different metrics to quantify the average emotional state of a user during a day (see Text S1): the fraction of posts expressing positive emotions (“positive rate”); and the fraction expressing negative emotions (“negative rate”). Note that the positive and negative emotions are not two ends of the same scale. Some messages will express both positive and negative emotions just as individuals experience mixed emotions on occasion, so it is possible to score high on both measures. We then aggregate individual observations by city and day, restricting our attention to all English-speaking Facebook users residing in the 100 most populous US cities.

## Results

**Figure 1:**
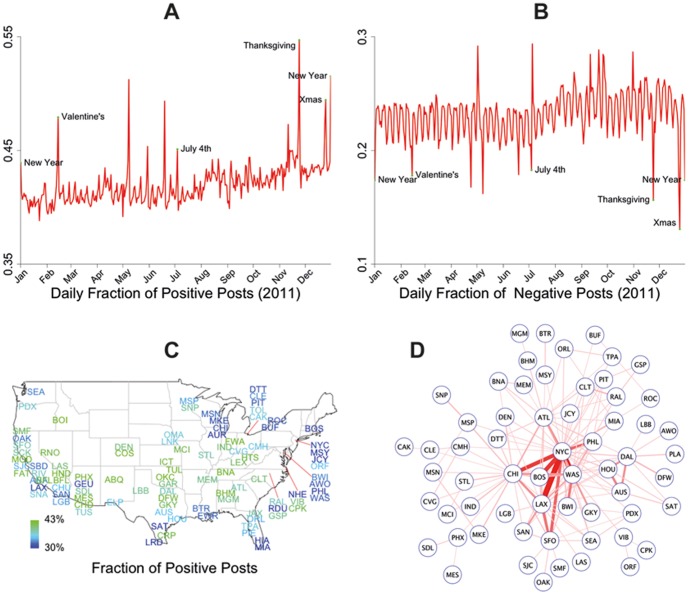
Description of the data. Temporal and geographic variation in emotions expressed by Facebook users in 2011 as measured by (a) the fraction of status updates containing positive emotion words; (b) the fraction of status updates containing negative emotion words. Extreme values are noted for holidays. (c) A map of the U.S. with approximate locations of the 100 most populous cities (represented by airport code) and their average fraction of posts with positive emotions (blue is less and green is more). (d) Network of between-city ties for all pairs of cities with at least 50,000 friendships. Darker, thicker lines indicate more friendship ties (maximum = 1,210,769).

Consistent with recently-published research on Twitter posts [28], Fig. 1 shows temporal patterns of variation in positive and negative emotions on Facebook that correspond to greater happiness on weekends and holidays. Fig. 1 also shows geographic variation in emotion expression and illustrates the number of between-city friendships in larger cities.

**Figure 2:**
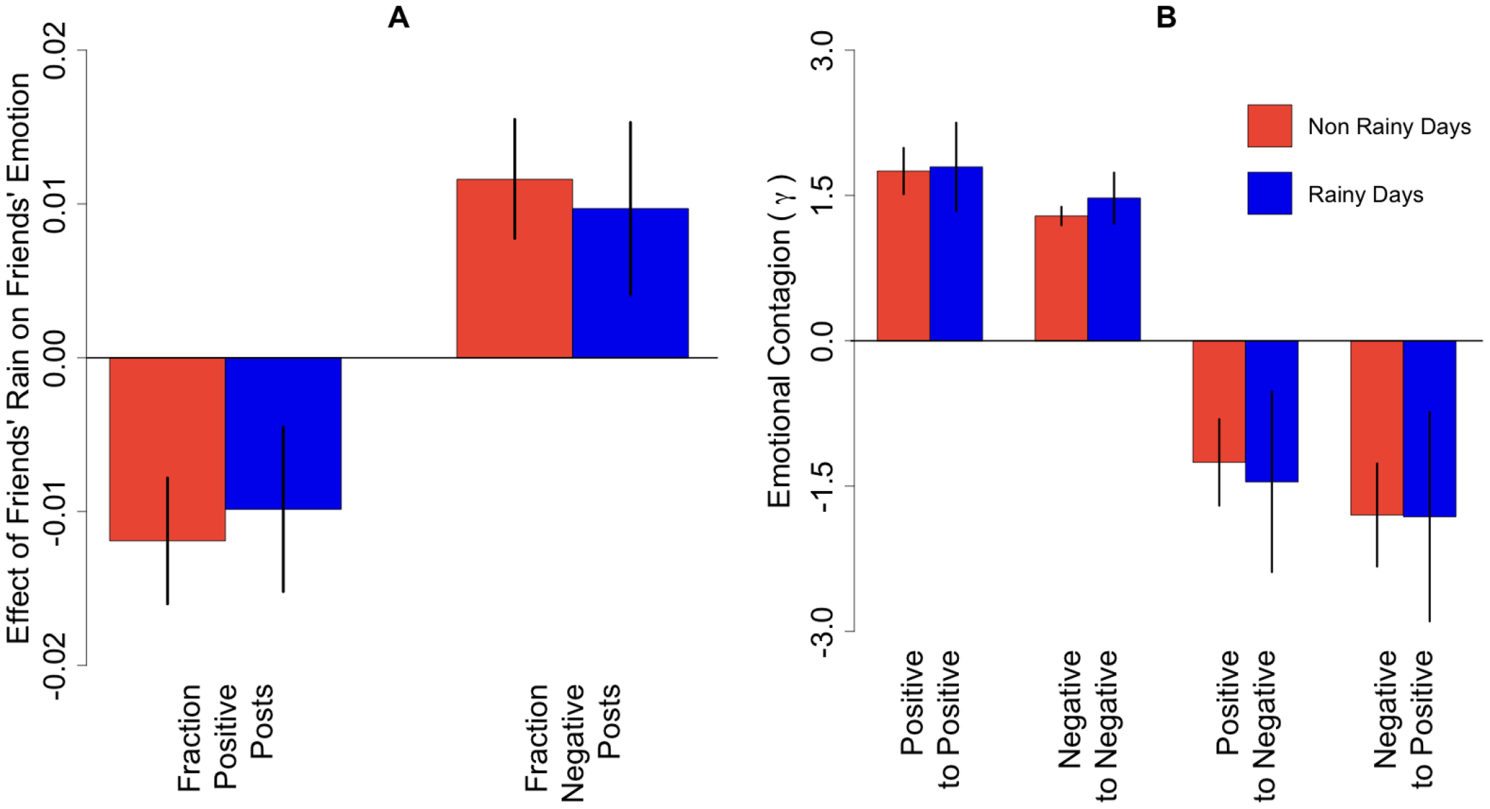
Model estimates. (a) Difference in emotional expression between days with and without rain. Estimates derived from first stage regressions of each measure of emotion on a binary measure of rainfall. (b) Estimates of emotional contagion between friends from the second stage of an instrumental variables regression from four separate models. The results show that rain affects emotional expression, both positive and negative posts are contagious, and positive posts tend to inhibit negative posts and vice versa. All models include fixed effects for city and day, average friends' weather in other cities, and standard errors clustered by city and day (see Text S1). Vertical bars show 95% confidence intervals.

We matched these observations to publicly available meteorological records that indicate total precipitation for each day in each of these cities. Fig. 2a shows results from the “first stage” regressions that estimate the effect of rainfall on a user's emotion. We find that an average rainy day decreases the number of positive posts by 1.19% (95% CI: 0.78% to 1.60%) and also increases the number of negative posts by 1.16% (0.78% to 1.55%). While these effects are small, it is their statistical significance – not size – that matters, since the goal is to use them as instruments to study the effect of exogenous variation in friends' emotional expression on one's own expression. Both models generate test statistics that suggest the rainfall instruments are strong enough to provide adequate power and that they are also appropriately identified (see Text S1).

Given widespread folk beliefs about the effect of mood on weather, it is perhaps somewhat surprising that this relationship is contested. Past research has generally focused on small samples and researchers have argued that inconsistent results mean the effect of rainfall is contingent on season [29] or personality type [30], but the results here suggest that the reason for the inconsistent results may be due to lack of power. Another recent (and highly powered) study of Twitter data also uses weather variables to improve predictive power in a model of sentiment, but the researchers do not separate the effect of rainfall from other weather variables [31].

Using predicted variation in emotional expression based on the exogenous effect of rainfall, we can now estimate the total effect of a user on all her friends, which is quantified by the contagion variable *γ* (see Text S1 for proof). Fig. 2b shows that each additional positive post yields an additional 1.75 (95% CI 1.51 to 1.99) positive posts amongst one's friends. Meanwhile, each additional negative post yields 1.29 (95% CI 1.19 to 1.38) more negative posts by friends. In other words, the total effect of rainfall on emotional expression is about 150% larger than we would expect if we were only measuring the direct effect on users and ignoring the indirect effect on their friends. And intriguingly, although rain is the impetus for this contagion, positive messages appear to be more contagious than negative (*p* = 0.001 for the comparison).

Fig. 2b also shows that positive and negative emotional expressions tend to have an inhibitory effect on one another. Each additional positive post *decreases* the number of friends' negative posts by 1.80 (95% CI 1.27 to 2.33), and each additional negative post decreases the number of friends' positive posts by 1.26 (95% CI 0.81 to 1.70). Again, positive messages appear to have a stronger effect, though here the difference is not significant (*p* = 0.12) and therefore may be due to chance.

We also evaluated these models when we restricted observations to rainy days (rather than restricting them to non rainy days) and found that rainy days elsewhere have just as strong an effect in all cases, regardless of the weather a person experiences directly (see Fig. 2b).

**Figure 3:**
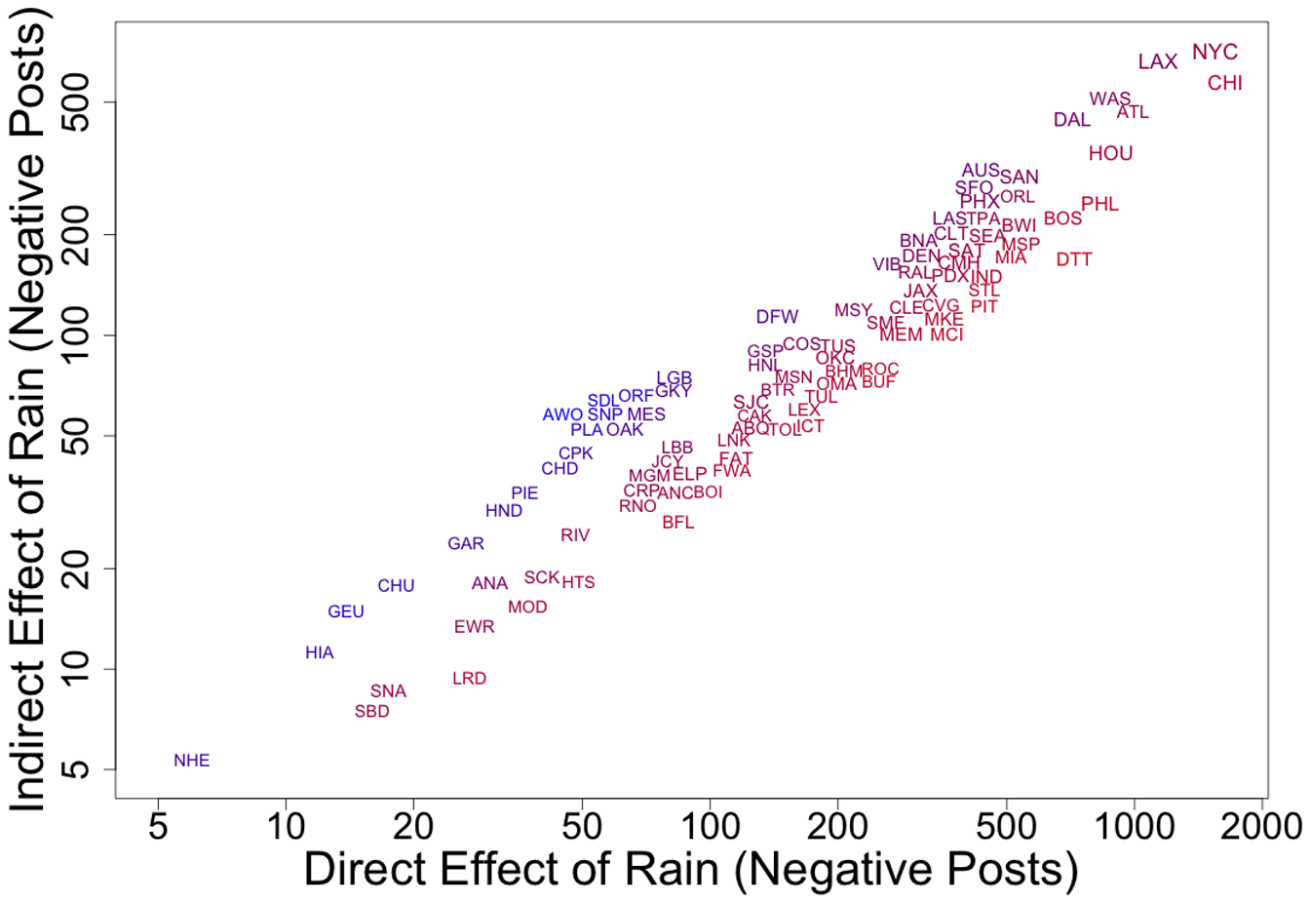
Predicted effects. Total number of negative posts generated by a day of rainfall within a city (direct) and in other cities via contagion (indirect). Blue colors indicate higher indirect/direct effect ratio. Larger labels indicate larger population.

Our model allows us to measure the total direct effect of rainfall on the number of positive and negative posts in each city, which is an increasing function of the number of users. We can also measure the total *indirect* effect of rainfall in one city on users in other cities, which is an increasing function of the number of users and their average number of friends in other cities, but a decreasing function of the friends of those friends (since people with more friends are less likely to be influenced by any one of them in particular). For example, we estimate that a rainy day in New York City directly yields an additional 1500 (95% CI 1100 to 2100) negative posts by users in New York City and about 700 (95% CI 600 to 800) negative posts by their friends elsewhere. Fig. 3 shows results like these for all 100 cities in our sample (see Text S1 for details and confidence intervals).

To evaluate the robustness of our method for estimating emotional contagion, we created a “placebo” test of the effect of *future* weather and resulting emotional expression on *today's* emotional expression by friends. In Text S1, we show that none of our four models generates a significant estimate for contagion that travels backwards in time. In future work, it may be helpful to have greater resolution for the time of the exogenous factor to see how the effect of emotional contagion changes over hours or minutes. Moreover, to exclude the possibility that the emotion contagion we measure is merely topic contagion between people writing posts about the weather, we conducted additional tests that control for the frequency of weather-related posts. In all cases, the estimates for the social contagion effect *γ* are substantially the same as in the original model, suggesting that the results are not driven by topic contagion (see Text S1).

## Discussion

Our estimates of the social contagion of emotional expression suggest that there may be large-scale spillovers in online networks. What people feel and say in one place may spread to many parts of the globe on the very same day. Yet the 1.5∶1 estimated ratio of the indirect to the direct effect is actually somewhat lower than other kinds of network effects measured experimentally. For the spread of giving behavior in a public goods experiment, for example, it is estimated that each dollar given yielded two dollars in giving by others [32]. For voting behavior, a recent large-scale experiment suggested the ratio is about four to one [17].

While the method we elaborate here is potentially applicable to a wide variety of emotions and behaviors online, an important limitation is that we cannot use this method to estimate contagion effects *within* subpopulations. It is plausible that these effects might be even stronger when subpopulations are geographically defined (as in cities), since many studies suggest that physical proximity increases social influence between connected individuals [16]. Another limitation is that instruments are not always readily available, and in some cases it may be unclear whether they are causally and directly related to the outcome variable of interest. However, when such instruments are indeed available, this approach may prove to be a useful alternative to costly large-scale experiments with limited external validity since they require neither experimental control nor alteration of the user environment.

Although there are many factors that affect human emotions [33], [34], [35], we have confirmed here that individual expression of emotions depends on what others in an individual's social network are expressing. These results imply that emotions themselves might ripple through social networks to generate large-scale synchrony that gives rise to clusters of happy and unhappy individuals. And new technologies online may be increasing this synchrony by giving people more avenues to express themselves to a wider range of social contacts. As a result, we may see greater spikes in global emotion that could generate increased volatility in everything from political systems to financial markets [36].

Our results are also consistent with prior work on the evolutionary basis of human emotions and with prior work focusing on the fleeting, direct spread of emotions. In addition to their internal and psychological relevance [37], emotions play a specifically social role: when humans experience emotions, they do not generally keep them to themselves, but rather, they tend to *show* them. Like laughter and smiling [38], emotions expressed online may serve the evolutionarily adaptive purpose of enhancing social bonds. Human laughter, for example, is believed to have evolved from the “play face” expression seen in other primates in relaxed, social situations [39]. Such facial expressions and positive emotions enhance social relations by producing analogous pleasurable feelings in others [16], by rewarding the efforts of others, and by encouraging ongoing social contact. Given the organization of people (and early hominids) into social groups larger than pairs [40], such spread in emotions probably served evolutionarily adaptive purposes. In this regard, it is noteworthy that, during our study period, users were increasingly expressing emotions as they adapted to Facebook as a new communication environment.

Our findings also have significance for public wellbeing. To the extent that clinical or policy maneuvers increase the happiness of one person, they may have cascade effects on others in their social networks, thereby enhancing the efficacy and cost-effectiveness of the intervention, and these results suggest that such cascade effects may be promoted online. For example, providing better care for those who are suffering might not only improve their happiness, but also the happiness of numerous others, thereby further vindicating the benefits of medical care or public policy.

## Supporting Information

Text S1 This document contains the detailed derivation of the model, description of estimation techniques, exposition of results and robustness tests.
(PDF)
