# Supplementary material for: Detecting Emotional Contagion in Massive Social Networks
Source: PLoS One. 2014 Mar 12;9(3):e90315. doi: 10.1371/journal.pone.0090315 (PMC3951248; doi:10.1371/journal.pone.0090315)
Supplement: Text S1 — This document contains the detailed derivation of the model, description of estimation techniques, exposition of results and robustness tests. (PDF) [file pone.0090315.s001.pdf]

Supporting Information for

Detecting Emotional Contagion in Massive Social Networks

Lorenzo Coviello, Yunkyu Sohn, Adam D.I. Kramer,
Cameron Marlow, Massimo Franceschetti,
Nicholas A. Christakis, James H. Fowler

Contents

1 A model of emotional contagion **3**

2 Aggregating the model **5**

3 Data **6**

4 Variables of the model **7**

5 Model estimation **9**

6 Quantifying the total effect of a user on her friends **16**

7 How rain affects friends in other cities **17**

8 Tables **20**

Bibliography **38**

1 A model of emotional contagion

Let y_{it} be the emotional expression of individual i at time t. Let a_{ijt} be the strength of the relationship from individual i to individual j at time t. Note that a_{ijt} need not be symmetric (i may perceive a stronger relationship with j than j does with i), and it allows for temporal variations. Let $\delta_{it} = \sum_j a_{ijt}$ be the degree of individual i at time t.

In the simplest case, a_{ijt} can take binary values, 1 designating that a relationship between i and j exists at time t, 0 designating that it does not. Under this assumption, δ_{it} is simply the number of her social contacts of i at time t.

Suppose there are three kinds of exogenous factors that affect emotion. First, there are factors that are time-varying and affect everyone equally (like holidays, for example). We denote these with a fixed effect θ_t for each time period t. Second, there are factors that are time-invariant and specific to an individual (such as a person's baseline personality). We denote these with a fixed effect f_j for each individual j. Third, some factors are *both* time-varying and specific to an individual (like the weather). We denote these by x_{jt} for each individual j and time period t.

In addition, suppose there is an endogenous factor that affects the emotion of each individual in proportion to the strength of the relationship between j and her social contacts. That is, each individual j is affected by the specific emotion on day t of each individual i to whom she is connected.

Assuming a memoryless model where individuals influence each other only within a time period t and not across time periods, we can specify a *linear model* for the emotion y of individual j on day t:

$$y_{jt} = \theta_t + f_j + \beta x_{jt} + \gamma \frac{1}{\delta_{jt}} \sum_i a_{ijt} y_{it} + \epsilon_{jt}, \qquad (1)$$

where β indicates the strength and direction of influence of the time-varying exogenous factor, γ indicates the strength and direction of social influence, and ϵ_{jt} is a normally distributed error term with mean zero and variance σ^2 that is independent and identically distributed (i.i.d.) across both individuals and time.

Observe that the model in equation (1) assumes that influence is averaged over all social contacts and therefore inversely proportional to the cumulative weight δ_{jt} of all j's relationships. If a_{ijt} takes binary values, then this implies that the influence from i to j is inversely proportional to the number of j's social contacts. This assumption is based on the idea that an individual with many social contacts is less likely to be influenced by each single contact i than an individual with few social contacts.

We are interested in estimating the value of the influence factor γ, which is difficult due to the inherent *feedback* present in the process of emotional contagion. Correlation in emotions may not only the result from pairwise mutual influence, but also from cycles in the social network. For example, i might influence k's emotional expression, which in turns affects j's emotional expression, and so on. We address the inherent endogeneity of contagion in Section 5 by using instrumental variable regression [2].

A second difficulty here is the large size of our data set. We would like to apply our model to the longitudinal content generated by millions of users with billions of friends over hundreds of days. We address this difficulty in Section 2, where we propose a method to estimate the individual-level parameter γ using aggregated data. A key to this method is to identify a unit of analysis in which many individuals within the same subpopulation are affected by the same exogenous variables. For example, individuals i and j may be in the same city g and therefore experience the same weather, traffic conditions, sporting event outcomes, and so on. Or they may be in different cities g and h, in which case their different exposures to exogenous factors may help us to identify how one person affects another. In our aggregated model, we leverage these between-unit social ties to consider how a factor in city g affects individual i, which in turn affects individual j who was not exposed directly to that factor because she is in city h. In other words, if it rains on you in New York, does it make your friends in San Diego less happy?

2 Aggregating the model

The model in equation (1) can be computationally demanding in big data sets, since there is one observation for each individual-time pair. We therefore simplify the model further by averaging equation (1) over all n_g individuals in a given subpopulation S_g who are in city g.

$$\frac{1}{n_g}\sum_{j\in S_g} y_{jt} = \frac{1}{n_g}\sum_{j\in S_g}\left(\theta_t + f_j + \beta x_{jt} + \gamma\frac{1}{\delta_{jt}}\sum_i a_{ijt}y_{it} + \epsilon_{jt}\right). \tag{2}$$

We can change the notation to make things clearer.

Let $\bar{y}_{gt} = \frac{1}{n_g}\sum_{j\in S_g} y_{jt}$ be the average emotion at time t for all individuals in subpopulation S_g.

Let $\bar{f}_{gt} = \frac{1}{n_g}\sum_{j\in S_g} f_j$ be the average individual fixed effects for all individuals in subpopulation S_g (this is therefore a city-level fixed effect).

Let $\bar{x}_{gt} = \frac{1}{n_g}\sum_{j\in S_g} x_{jt}$ be the average exogenous variable at time t for all individuals in subpopulation S_g (this is therefore a city-level exogenous variable).

Let $\bar{Y}_{gt} = \frac{1}{n_g}\sum_{j\in S_g}\frac{1}{\delta_{jt}}\sum_i a_{ijt}y_{it}$. We can exchange the ordering of the summations and write

$$\bar{Y}_{gt} = \sum_i y_{it}\frac{1}{n_g}\sum_{j\in S_g}\frac{1}{\delta_{jt}}a_{ijt}$$

Observe that the term $\frac{1}{n_g}\sum_{j\in S_g}\frac{1}{\delta_{jt}}a_{ijt}$ represents the average strength of the relationship between i and an individual in city g. Therefore, $\bar{Y}_{gt}$ represents the average emotional influence at time t on an individual in city g.

The model in equation (2) can now be written as

$$\bar{y}_{gt} = \theta_t + \bar{f}_g + \beta\bar{x}_{gt} + \gamma\bar{Y}_{gt} + \bar{\epsilon}_{gt} \tag{3}$$

where $\bar{\epsilon}_{gt} = \frac{1}{n_g}\sum_{j\in S_g}\epsilon_{jt}$ is a city-specific error for all individuals j who are in city g. Since the error is a mean of normally distributed variables,

$\bar{\epsilon}_{gt}$ will also be normally distributed with mean 0, but it will have a city-specific variance σ^2/n_g. Notice that this indicates the variance is inversely proportional to the number of individuals in a city. As we describe below, we can use the equation for the variance explicitly to weight each observation in the model.

3 Data

Our period of observation starts on January 1st 2009 and ends on March 31st 2012, for a total of 1185 consecutive days. Data for five days of 2009 (March 4th, June 24th, August 15th, September 13th, November 11th) was not available at the time of analysis, so we consider the remaining 1180 days.

Data were collected from the Facebook online social network, and data were analyzed in aggregate within Facebook's data centers. Researchers did not access any personal information.

For each day in the period of observation, we consider all Facebook users in the 100 most populous US cites, and their status updates. Table 1 reports the list of the cities, each paired with the corresponding three-letter code used in the figures (airport codes in most cases). In particular, the subpopulation of Facebook users in a given city contains all users that (i) chose English as the language in which they view the website, (ii) selected United States as Country in their profile settings, (iii) can be matched to city g by IP-based geographic location. We build separate user pools for different days to allow us to take user mobility into account, since on any particular day a user might travel or move to a new city.

For each Facebook user, we measured emotion using all status updates as explained in the next section. We used only status updates, which can be viewed as personal self-expression, and did not consider more directed forms of communication on Facebook (e.g., chat, private messages, comments). We also measured social contacts for each day in the observation period, letting $a_{ijt} = 1$ for all pairs of users i and j who were "friends" with one another on day t, and 0 otherwise.

Table 2 summarizes our sample size by showing mean and standard deviation of the daily number of users, number of status updates, and friendship ties.

4 Variables of the model

4.1 Emotion variables

For user i and day t, let U_{it} be the set of status updates posted by i on day t, and let $u_{it} = |U_{it}|$ be its cardinality. Let $u_{it}^{(p)}$ be the number of status updates in U_{it} that contain at least one word from the "positive emotion" category defined by LIWC 2007 [1]. Similarly, let $u_{it}^{(n)}$ be the number of status updates in U_{it} that contain at least one word from the "negative emotion" category.

Note that a single status update might contain both a negative word and a positive word, therefore contributing to both $u_{it}^{(p)}$ and $u_{it}^{(n)}$. Moreover, our analysis simply considers raw matching of positive and negative words, without making any attempt to identify expressions like negations or sarcasm.

We measure emotion in two ways based on these definitions: (i) the rate of status updates that contain words, (ii) the rate of status updates that contain negative words.

Consider a user i and a day t such that $u_{it} \neq 0$. The positive rate of user i on day t is defined as

$$y_{it}^{(p)} = \frac{u_{it}^{(p)}}{u_{it}},$$

that is, the fraction of status updates with at least one positive word. Note that $0 \leq y_{it}^{(p)} \leq 1$.

Similarly, the negative rate of user i on day t is defined as

$$y_{it}^{(n)} = \frac{u_{it}^{(n)}}{u_{it}},$$

that is, the fraction of status updates with at least one negative word. Note that $0 \leq y_{it}^{(n)} \leq 1$.

By averaging these quantities over all users in city g, we obtain the average positive rate and negative rate of that city. Let S_g be the set of n_g users i in city g such that $u_{it} \neq 0$. That is,

$$\bar{y}_{gt}^{(p)} = \frac{1}{n_g} \sum_{i \in S_g} y_{it}^{(p)},$$
$$\bar{y}_{gt}^{(n)} = \frac{1}{n_g} \sum_{i \in S_g} y_{it}^{(n)},$$

Table 3 shows mean values for each of these emotion variables.

The variables $\bar{Y}_{gt}^{(p)}$ and $\bar{Y}_{gt}^{(n)}$ of the model in equation (3) are given by

$$\bar{Y}_{gt}^{(p)} = \sum_{i} y_{it}^{(p)} \frac{1}{n_g} \sum_{j \in S_g} \frac{1}{\delta_{jt}} a_{ijt},$$
$$\bar{Y}_{gt}^{(n)} = \sum_{i} y_{it}^{(n)} \frac{1}{n_g} \sum_{j \in S_g} \frac{1}{\delta_{jt}} a_{ijt}.$$

4.2 Meteorological variables

For each day in the period of observation, meteorological data for the 100 US cities under observation were made available by the US National Climatic Data Center (NCDC, http://www.ncdc.noaa.gov). For each city, we consider the data from the NCDC station closest to the airport or to the city center.

For each city g and day t we consider a binary indicator variable $\bar{x}_{gt}$ equal to 1 if it rained in city g on day t and equal to 0 otherwise. Table 4 shows the number of rainy days in each city, during the period of observation.

For the instrumental variable regression described below we will make use

of the variable

$$\bar{X}_{gt} = \sum_i x_{it} \frac{1}{n_g} \sum_{j \in S_g} \frac{1}{\delta_{jt}} a_{ijt}. \tag{4}$$

In particular, if user i is in city h then $x_{it} = \bar{x}_{ht}$ (the user's own weather is the same as the average weather of all users in the same city).

5 Model estimation

We are interested in estimating the parameters of the model in equation (3), which is simply an aggregated restatement of the individual-level model in equation (1). To recap, this model is:

$$\bar{y}_{gt} = \theta_t + \bar{f}_g + \beta \bar{x}_{gt} + \gamma \bar{Y}_{gt} + \bar{\epsilon}_{gt},$$

and we are primarily interested in estimating the effect of emotional contagion (γ). The dependent variable $\bar{y}_{gt}$ is the average emotion of users in city g on day t, the independent variable $\bar{Y}_{gt}$ is the average emotion of the friends of these users, $\bar{x}_{gt}$ is a binary indicator variable for rainfall in city g, and θ_t and $\bar{f}_g$ are fixed effects for each day and each city.

Note that we can estimate γ for contagion of either positive and negative emotion, and we can also see if these two emotions tend to inhibit one another by estimating the effect of friends' positive emotion on users' negative emotion and vice versa.

An observation period of 1180 days and a set of 100 cities results in a model with $118,000$ observations, each corresponding to a city-day pair. The parameters that need to be estimated are the coefficients β and γ, 1180 fixed effects for the days, and 100 fixed effects for the cities.

Since one of the explanatory variables of the model in equation (3), $\bar{Y}_{gt}$, is an endogenous variable (i.e. it is correlated both to the dependent variable $\bar{y}_{gt}$ and to the error term $\bar{\epsilon}_{gt}$), ordinary least squares regression would yield biased coefficient estimates. We therefore use *instrumental variable regression* [2].

Instrumental variable regression is an estimation method that can produce consistent and unbiased estimates when one of the explanatory variables is correlated with the error terms in the model equation. This is the case when there is *reciprocal causality* from the dependent variable to an explanatory variable (in our case, users affect their friends and vice versa), when one or more relevant explanatory variables are omitted from the model, or when the covariates are affected by measurement errors. If an *instrument* is available that predicts the endogenous variable, then consistent and unbiased estimates can be obtained. In a linear model, an instrument for an endogenous explanatory variable v is a variable z that does not appear in the model equation, is correlated with v (conditional on all the exogenous explanatory variables) and is not correlated with the error term. [2]

In our model, an instrument for the endogenous explanatory variable $\bar{Y}_{gt}$ is an exogenous variable z that is not correlated to the error term in equation (3), that is $Cov(z, \hat{\epsilon}_{gt}) = 0$, and is partially correlated to $\bar{Y}_{gt}$ when controlling for the other exogenous explanatory variables. In the context of our model, we can write:

$$\bar{Y}_{gt} = \theta'_t + \bar{f}'_g + \beta_2 \bar{x}_{gt} + \beta_1 z + \nu_{gt}, \tag{5}$$

where ν_{gt} is an error term not correlated to any regressors and θ'_t and $\bar{f}'_g$ are separately estimated time and subpopulation fixed effects.

Equation (5) can be seen as the linear projection of $\bar{Y}_{gt}$ on the space of all the exogenous variables. Substituting equation (5) into equation (3) yields:

$$\bar{y}_{gt} = (\theta_t + \gamma\theta'_t) + (\bar{f}_g + \gamma\bar{f}'_g) + (\beta + \gamma\beta_2)\bar{x}_{gt} + \gamma\beta_1 z + \bar{\epsilon}'_{gt}, \tag{6}$$

where the error term is uncorrelated with all the explanatory variables.

We use the variable $\bar{X}_{gt}$ defined in equation (4) as the instrument (z) for $\bar{Y}_{gt}$, as it is uncorrelated with the error term in equation (3) and it is partially correlated to $\bar{Y}_{gt}$ (see Tables 5 to 8 for details). Specifically, we utilize rainfall experienced by the friends of users in city g to predict the emotion of those friends since it directly affects their mood.

The procedure above is equivalent to estimating the model in equation (3) using two stage least-squares (2SLS) regression. The first stage regression estimates a model of the form

$$\bar{Y}_{gt} = \theta'_t + \bar{f}'_g + \beta_1 \bar{X}_{gt} + \beta_2 \bar{x}_{gt} + \epsilon'_{gt}. \tag{7}$$

The second stage regression uses the predicted values $\bar{Y}_{gt}^{pred}$ from the first stage to estimate the model

$$\bar{y}_{gt} = \theta_t + \bar{f}_g + \beta \bar{x}_{gt} + \gamma \bar{Y}_{gt}^{pred} + \bar{\epsilon}_{gt}. \tag{8}$$

Finally, recall that the variance of the $\bar{\epsilon}_{gt}$ error term is proportional to $\frac{1}{n_g}$ where n_g is the number of individuals in a city. We therefore weight each observation by the corresponding value of n_g.

A key assumption of instrumental variables regression is the exclusion restriction – the instrument must not directly influence the dependent variable. In our case, some of the users' friends are experiencing the same weather as the users because they are in the same city. Therefore, in order to break any possible correlation between friends' rainfall $\bar{X}_{gt}$ and users' rainfall $\bar{x}_{gt}$, we only consider observations for city-day pairs (g, t) such that $\bar{x}_{gt} = 0$ (that is, it did not rain in city g on day t). This results in dropping $30,300$ observations, for a total of $87,700$ remaining observations. Conditional on $\bar{x}_{gt} = 0$, equations (7) and (8) can be respectively written as

$$\bar{Y}_{gt} = \theta'_t + \bar{f}'_g + \beta_1 \bar{X}_{gt} + \epsilon'_{gt}, \tag{9}$$

$$\bar{y}_{gt} = \theta_t + \bar{f}_g + \gamma \bar{Y}_{gt}^{pred} + \bar{\epsilon}_{gt}. \tag{10}$$

Note that since $\bar{x}_{gt} = 0$, there is no rainfall for either the user or the user's friends who are in the same city. This means that the instrument $\bar{X}_{gt}$ now depends *only on friends who are in different cities* (not in city g).

Tables 5 to 8 report the estimates (with standard errors, t-statistics, 95% confidence intervals, and diagnostic statistics) for the first and second stage of the 2SLS regression for the model in equation (3) (fixed effects estimates are not reported due to their number). The estimates of the emotional transmission parameter γ from the second stage regression are always significantly different than zero, and the two positive coefficients support the hypothesis of contagion. When users' friends post positive status updates, it increases their own positive updates. When users' friends post negative status updates, it increases their own negative updates. At the same time, the two negative coefficients for γ support the idea that opposite moods have an inhibitory effect. When users' friends post positive status updates, it decreases their own negative updates. When users' friends post negative status updates, it decreases their own positive updates.

In order to assess the quality of the estimates obtained via instrumental variable regression, we also compute diagnostic statistics.

First, we need to verify that the model is not underidentified. The Kleinbergen-Paap *rk* LM statistic allows to test the null hypothesis of underidentification [3], and all of our tests reject the null (test statistics are reported in the caption underneath each table).

Second, we need to verify that the instruments are good predictors of the endogenous explanatory variable in the first-stage regression (otherwise the instruments are considered *weak*). Weak instruments would cause poor predicted values in the first-stage regression (for example, little variation) and presumably poor estimation in the second-stage regression. To ensure the instruments are not weak, the Cragg-Donald Wald F statistic must exceed the critical threshold suggested by Stock and Yogo [5].

For robustness, we also tested a version of the model in equation (3) that only considers observations for city-day pairs (g, t) such that $\bar{x}_{gt} = 1$ (that is, it rained in city g on day t). This results in dropping $87,700$ observations, for a total of $30,300$ remaining observations. Tables 9 to 12 report the estimates (with standard errors, t-statistics, 95% confidence intervals, and diagnostic statistics) for the first and second stage of the 2SLS regression, which are substantially the same as the ones in Tables 5 to 8, with overlapping 95% CI. These results suggest that the users' own experience of rainfall does not affect emotional contagion online.

5.1 Simulation with Synthetic Data

Before applying the estimation strategy to empirical data, we run simulation to examine the validity of our approach. We generate synthetic data on a 2-dimensional geographical space in a manner to incorporate realistic properties of meteorological data and friendship networks. For simplicity, we assume invariant equilibrium behavior at a single snapshot observation. Each individual is located on a vertex of a grid on the geographical space, $(d_{1i}, d_{2i}) \in [0, 1] \times [0, 1]$. The 1-dimensional exogenous variable x is defined continuously over the geographical space drawn from

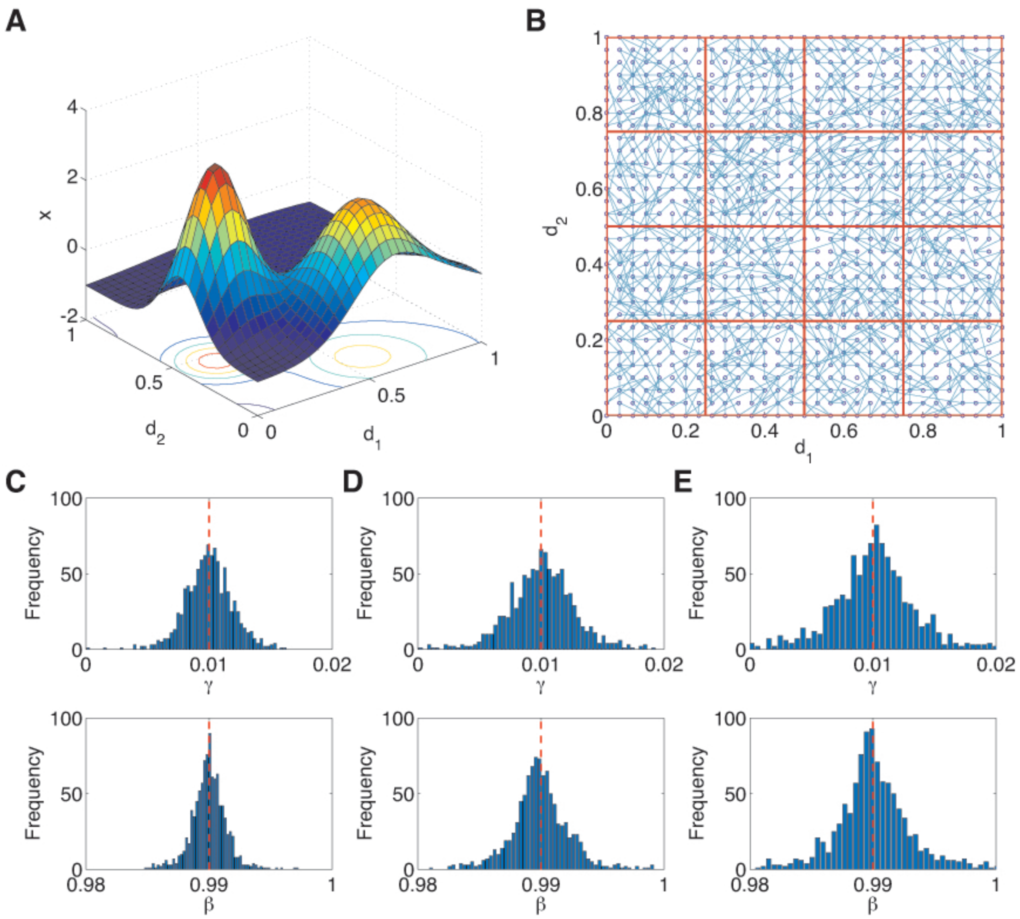

Fig. S1: (A) The exogenous variable x is a z-scored Gaussian mixture distribution defined over the 2-dimensional geographical space. (B) Each individual is located on a vertex of a grid structure in the geographical space. For an individual i who is located on (d_{1i}, d_{2i}), $x_i = x(d_{1i}, d_{2i})$. Each circle represents an individual and the blue lines between individuals are existing social ties in A. Red lines are borders of groups. (C-E) Each histograms is the frequency distribution of γ and β obtained from 2SLS regression throughout 1,000 trials of simulation. Red dashed lines indicate true values of coefficients. To ensure the robustness of the results, we vary the distant-dependency level α of A while preserving the connection density of the networks by adjusting ρ. The ratio of the number of existing links to the number of all possible connections, connection density, is set to ~ 0.02. $N = 961$ and $n = 100$. $\alpha = 16$ (C), $\alpha = 26$ (D) and $\alpha = 36$ (E). The extent of geographical clustering also affects the level of topological clustering measured by average clustering coefficient, the ratio of the number of connected triads to the number of all possible combinations. Average clustering coefficient of the networks grows from 0.14 to 0.37 as α increases.

a multidimensional Gaussian mixture distribution. The value of x affecting individual i corresponds to x at i's location, $x_i = f(x; d_{1i}, d_{2i})$ where $f(x; d_1, d_2) = \sum_{j=1}^{10} \mathcal{N}(\boldsymbol{\mu_j}, \boldsymbol{\Sigma_j})$ and $\boldsymbol{\mu_j} \in d_1 \times d_2$ and $\boldsymbol{\Sigma_j} = cI_2$ share the same geographical space with the individuals. $\boldsymbol{\mu_j}$ is randomly drawn from a 2-dimensional uniform distribution defined over $[0,1] \times [0,1]$ and c is drawn from a uniform distribution defined over $(0, 0.2)$. $N \times N$ adjacency matrix A is an undirected binary network generated by using a stochastic distant-dependent attachment model [4]. The probability that individual i projects an undirected link to another individual j is $\rho e^{-\alpha D_{ij}}$ where D_{ij} is the Euclidean distance between i and j. Non-negative coefficient α is a factor determining the level of distance dependency and coefficient $\rho \in [0,1]$ determines the density of the resulting network. As α increases the network is more likely to have proximal connections than distant connections.

We simulate the endogenous variable $\mathbf{y}$ using $\mathbf{x}$, A and the model equation (1). With a little calculation, we obtain the explicit expression $\mathbf{y} = (I - \gamma W)^{-1}(\beta \mathbf{x} + \epsilon)$ where $W_{ij} = \frac{a_{ij}}{\delta_i}$ (there is a slight notational change due to the matrix and vector representation). ϵ is drawn from $\mathcal{N}(0, 0.01^2)$. Ground truth value of γ is set to 0.01 and, for simplicity, $\beta = 1 - \gamma = 0.99$. Our challenge now is to recover β and γ using equations (7) and (8) by observing $\mathbf{y}$, $\mathbf{x}$ and A. Each individual is assigned to one of n ($n < N$) groups whose size is about the same to each other.

Figure S1 shows that 2SLS regression using equations (7) and (8) accurately recover the true values of coefficients in most cases. For networks with different level of geographical and topological clustering, we conduct 1,000 simulations for each setting. Standard deviation of coefficients in the pooled data is 0.0027 (γ) and 0.0024 (β) respectively around the true values. In total, more than 95% of individual coefficient estimates exhibit $p < 0.01$.

5.2 Placebo test

If our procedure is correctly estimating social influence, we would not expect to be able to predict users' emotion using future friends' weather and emotion. Here, we test a placebo model by using the same instrumental variables procedure described above to estimate the effect of *future* friends'

rainfall on users' emotion *today*. We arbitrarily choose $t+30$ as a point in time far enough in the future that friends' rainfall then will not be correlated with friends' rainfall at time t. We then modify the equation in (3) to shift the independent variable forward by 30 days:

$$\bar{y}_{gt} = \theta_t + \bar{f}_g + \beta \bar{x}_{gt} + \gamma \bar{Y}_{g,t+30} + \bar{\epsilon}_{gt}, \tag{11}$$

We conduct instrumental variable regression using $\bar{X}_{g,t+30}$ as an instrument for $\bar{Y}_{g,t+30}$. Tables 13 to 16 report the estimates (with standard errors, t-statistics, 95% confidence intervals, and diagnostic statistics) for the first and second stage of the 2SLS regression. The estimates of γ from the second stage regression are not statistically significant and they are much lower in magnitude than those estimated for the model in equation (3).

5.3 Controlling for topic contagion

One concern is that our estimates of emotional contagion are actually estimates of topic contagion. Friends who post more negatively when it rains may be posting about the weather itself, and users may respond with their own statuses about weather. This would not undermine our statistical results on contagion, but it might change our interpretation if we discovered that topics were driving the similarity in word choice by users and friends.

To address this issue, we created a dictionary of weather terms based on a meteorological glossary supplied by NOAA (http://www.erh.noaa.gov/box/glossary.htm). We then crowdsourced this dictionary to approximately 100 students, post-docs, and professors asking for additional suggestions. The resulting list is not exhaustive, but we expect it will allow us to detect most status updates that are on a weather-related topic. The full list of terms can be found in Table 17.

Recall that U_{it} represents the status updates of user i on day t, and let $u_{it}^{(w)}$ be the number of status updates in U_{it} that contain at least one word from our dictionary of weather terms. If $u_{it} \neq 0$, let $w_{it} = u_{it}^{(w)}/u_{it}$ be the fraction of i's status updates related to weather, and let $\bar{w}_{gt} = \frac{1}{n_g}\sum_{i \in S_g} w_{it}$ be the average over city g. We can now use this variable to control for the tendency

to post status updates about the weather by adding it to equation (3):

$$\bar{y}_{gt} = \theta_t + \bar{f}_g + \lambda \bar{w}_{gt} + \gamma \bar{Y}_{gt} + \bar{\epsilon}_{gt}. \tag{12}$$

Tables 18 to 21 report the estimates (with standard errors, t-statistics, 95% confidence intervals, and diagnostic statistics) for the first and second stage of the 2SLS regression. The negative estimates for λ suggest that increased usage of weather words is generally associated with decreased emotional expression. However, the relationships are weak and sometimes insignificant, and more importantly, the estimates for the emotional transmission parameter γ remain substantially the same as the estimates from model (3) without controls. These results indicate that posting on the topic of weather is not driving the relationship in use of emotional words between users and their friends.

6 Quantifying the total effect of a user on her friends

Consider a user j and assume she posts a single status update during day t. For presentation, we consider negative emotions and compare the case in which j's status update contains a negative word ($y_{jt} = 1$) versus the case it does not ($y_{jt} = 0$). We estimate the additional number of negative status updates posted by j's friends conditional on $y_{jt} = 1$ versus $y_{jt} = 0$.

According to the individual level model (1), the emotional contagion from j to i is given by $c_{ijt} = \gamma a_{ijt} y_{jt} / \delta_{it}$ for each user i who posted on day t (we assume that each user i posted either one or zero status updates). Conditional on $y_{jt} = 1$ and on $y_{jt} = 0$ respectively, this term is

$$c^{(1)}_{ijt} = \gamma a_{ijt} / \delta_{it},$$
$$c^{(0)}_{ijt} = 0.$$

Summing over all users i who posted on day t, the total emotional contagion of user j conditional on $y_{jt} = 1$ is

$$C^{(1)}_{jt} = \sum_i c^{(1)}_{ijt} = \gamma \sum_i a_{ijt} / \delta_{it},$$

while conditional on $y_{jt} = 0$ it is $C_{jt}^{(0)} = 0$. The difference in number of negative status updates posted by j's friends conditional on $y_{jt} = 1$ versus $y_{jt} = 0$ can be therefore quantified as

$$F_{jt} = C_{jt}^{(1)} - C_{jt}^{(0)} = \gamma \sum_i a_{ijt}/\delta_{it} = \gamma A_{jt},$$

where $A_{jt} = \sum_i a_{ijt}/\delta_{it}$ constitutes a measure of how influential user j is. In words, j's cumulative effect on her friends is proportional to the coefficient of emotional contagion γ and her influence A_{jt}.

Observe that A_{jt} can be computed exactly for each j and t. And note that this measure is increasing in the number of friends (more friends means more people might be influenced) and decreasing in the number of friends those friends have (if a friend has more friends, the user will on average have less influence on that friend).

The average user's effect $\bar{F}_t$ can be computed as the average individual effect over all n users

$$\begin{aligned}\bar{F}_t &= \frac{1}{n}\sum_j F_{jt} = \gamma\frac{1}{n}\sum_j A_{jt} = \gamma\frac{1}{n}\sum_j\sum_i a_{ijt}\frac{1}{\delta_{it}} \\ &= \gamma\frac{1}{n}\sum_i\frac{1}{\delta_{it}}\sum_j a_{ijt} = \gamma\frac{1}{n}\sum_i\frac{1}{\delta_{it}}\delta_{it} = \gamma.\end{aligned}$$

The average user's effect $\bar{F}_t$ and 95% CI for all four choices of emotions (user's positive/negative rate, friends' positive/negative rate) are shown in Table 22, and correspond directly to the estimates of γ in Tables 5 to 8. In other words, the γ coefficients themselves are estimates of the total effect a user has on all her friends.

7 How rain affects friends in other cities

Here we compute the cumulative effect that rain in one city has on all friends of users in that city who are in *different* cities. This allows us to answer the question: if it rains in New York, how many additional users in other cities post negative status updates as a result?

Consider the 2SLS model given by equations (9) and (10) for day t and city g,

$$\bar{Y}_{gt} = \theta'_t + \bar{f}'_g + \beta_1 \bar{X}_{gt} + \epsilon'_{gt},$$
$$\bar{y}_{gt} = \theta_t + \bar{f}_g + \gamma \bar{Y}^{pred}_{gt} + \bar{\epsilon}_{gt}.$$

Suppose that other cities are indexed by $h \neq g$ and let $\bar{X}^{(h,1)}_{gt}$, $\bar{Y}^{(h,1)}_{gt}$, $\bar{y}^{(h,1)}_{gt}$ respectively denote $\bar{X}_{gt}$, $\bar{Y}_{gt}$, $\bar{y}_{gt}$ conditional on $\bar{x}_{ht} = 1$ (that is, rainfall in city h). Similarly, let $\bar{X}^{(h,0)}_{gt}$, $\bar{Y}^{(h,0)}_{gt}$, $\bar{y}^{(h,0)}_{gt}$ be the same quantities conditional on $\bar{x}_{ht} = 0$. Using this notation, we can derive the following relationships:

$$\bar{X}^{(h,1)}_{gt} - \bar{X}^{(h,0)}_{gt} = \sum_{i \in S_h} \frac{1}{n_g} \sum_{j \in S_g} \frac{1}{\delta_{jt}} a_{ijt},$$
$$\bar{Y}^{(h,1)}_{gt} - \bar{Y}^{(h,0)}_{gt} = \beta_1 \left(\bar{X}^{(h,1)}_{gt} - \bar{X}^{(h,0)}_{gt} \right) = \beta_1 \sum_{i \in S_h} \frac{1}{n_g} \sum_{j \in S_g} \frac{1}{\delta_{jt}} a_{ijt},$$
$$\bar{y}^{(h,1)}_{gt} - \bar{y}^{(h,0)}_{gt} = \gamma\beta_1 \left(\bar{X}^{(h,1)}_{gt} - \bar{X}^{(h,0)}_{gt} \right) = \gamma\beta_1 \sum_{i \in S_h} \frac{1}{n_g} \sum_{j \in S_g} \frac{1}{\delta_{jt}} a_{ijt}.$$

Observe that $\bar{y}^{(h,1)}_{gt} - \bar{y}^{(h,0)}_{gt}$ is the difference in emotion of the average user in city g conditional on $\bar{x}_{ht} = 1$ verus $\bar{x}_{ht} = 0$. Assuming that each user posts either one or zero status updates on day t, $n_g(\bar{y}^{(h,1)}_{gt} - \bar{y}^{(h,0)}_{gt})$ is the additional number of negative status updates posted in city g conditional on $\bar{x}_{ht} = 1$ verus $\bar{x}_{ht} = 0$, where n_g is the number of users in city.

Fix a day t, let $\bar{I}_{ht}$ be the cumulative number of negative status updates posted in all cities different than h conditional on $\bar{x}_{ht} = 1$ versus $\bar{x}_{ht} = 0$, that is the *indirect* of rain in city h. This can be computed by summing the effect on each city $g \neq h$,

$$\bar{I}_{ht} = \sum_{g \neq h} n_g \left(\bar{y}^{(h,1)}_{gt} - \bar{y}^{(h,0)}_{gt} \right) = \gamma\beta_1 \sum_{g \neq h} n_g \sum_{i \in S_h} \frac{1}{n_g} \sum_{j \in S_g} \frac{1}{\delta_{jt}} a_{ijt}$$
$$= \gamma\beta_1 \sum_{i \in S_h} \sum_{g \neq h} \sum_{j \in S_g} \frac{1}{\delta_{jt}} a_{ijt} = \gamma\beta_1 \sum_{i \in S_h} \sum_{j \notin S_h} \frac{1}{\delta_{jt}} a_{ijt}.$$

For a user i in city h, $\sum_{j \notin S_h} a_{ijt}/\delta_{jt}$ is the sum of the inverse degrees of i's friend who are in a different city, and represents a measure of i's *influence*

outside city h. The indirect effect or rain $\bar{I}_{ht}$ is therefore proportional to the total influence from users in city h to their friends in other cities.

For each city h, we let $\bar{I}_h$ be the average of $\bar{T}_{ht}$ over all days t.

The confidence interval of $\bar{I}_h$ is computed from the confidence interval for the product $\gamma\beta_1$, as the other terms can be exactly computed. To compute a confidence interval on the product $\gamma\beta_1$ we cannot simply multiply the estimates of γ and β_1 in Table 6 derived from the model in equation (3) using two-stage least-squares regression, because they might be correlated We therefore use bootstrap sampling by independently generating 100 bootstrap samples of our data set. Each bootstrap sample is generated by first selecting 1180 days uniformly at random with replacement, and then selecting 100 cities uniformly at random with replacement for each of the 1180 selected days. For each bootstrap sample, we estimate the model in equation (3) using two-stage least-squares regression, and we compute the product between β_1 (from the first-stage regression) and γ (from the second-stage regression). We them compute the mean and 95% CI of the estimates of $\gamma\beta_1$ from all bootstrap samples.

Tables 23 and 24 show the *direct* effect $\bar{D}_h$ (with 95% CI) of rain in city h on status updates posted by users in that city, computed by multiplying the number of users n_h by the coefficient β_1 from the first stage in the 2SLS regression. The tables also show the *indirect* effect $\bar{I}_h$ (with 95% CI) for each city h of rain on status updates posted by users in other cities.

Similar results can be obtained by considering the effect on the number of either positive or negative posts, and using positive or negative emotions as the variable $\bar{Y}_{gt}$ in the first stage regression. The size and direction of $\bar{I}_h$ and $\bar{D}_h$ would depend on the magnitude and sign of $\gamma\beta_1$ and β_1 respectively.

8 Tables

List of US cities and codes					
Code	City	Code	City	Code	City
ABQ	Albuquerque, NM	GAR	Garland, TX	OKC	Oklahoma City, OK
ANA	Anaheim, CA	GEU	Glendale, AZ	OMA	Omaha, NE
ANC	Anchorage, AK	GKY	Arlington, TX	ORF	Norfolk, VA
ATL	Atlanta, GA	GSP	Greensboro, NC	ORL	Orlando, FL
AUR	Aurora, CO	HIA	Hialeah, FL	PDX	Portland, OR
AUS	Austin, TX	HND	Henderson, NV	PHL	Philadelphia, PA
AWO	Arlington, VA	HNL	Honolulu, HI	PHX	Phoenix, TX
BFL	Bakersfield, CA	HOU	Houston, TX	PIE	St Petersburg, FL
BHM	Birmingham, AL	HTS	Huntington, WV	PIT	Pittsburgh, PA
BNA	Nashville, TN	ICT	Wichita, KS	PLA	Plano, TX
BOI	Boise, ID	IND	Indianapolis, IN	RAL	Raleigh, NC
BOS	Boston, MA	JAX	Jacksonville, FL	RDU	Durham, NC
BTR	Baton Rouge, LA	JCY	Jersey City, NJ	RIV	Riverside, CA
BUF	Buffalo, NY	LAS	Las Vegas, NV	RNO	Reno, NV
BWI	Baltimore, MD	LAX	Los Angeles, CA	ROC	Rochester, NY
CAK	Akron, OH	LBB	Lubbock, TX	SAN	San Diego, CA
CHD	Chandler, AZ	LEX	Lexington, KY	SAT	San Antonio, TX
CHI	Chicago, IL	LGB	Long Beach, CA	SBD	San Bernardino, CA
CHU	Chula Vista, CA	LNK	Lincoln, NB	SCK	Stockton, CA
CLE	Cleveland, OH	LRD	Laredo, TX	SDL	Scottsdale, AZ
CLT	Charlotte, NC	MCI	Kansas City, MO	SEA	Seattle, WA
CMH	Columbus, OH	MEM	Memphis, TN	SFO	San Francisco, CA
COS	Colorado Springs, CO	MES	Mesa, CA	SJC	San Jose, CA
CPK	Chesapeake, VA	MGM	Montgomery, AL	SMF	Sacramento, CA
CRP	Corpus Christi, TX	MIA	Miami, FL	SNA	Santa Ana, CA
CVG	Cincinnati, OH	MKE	Milwaukee, WI	SNP	St Paul, MN
DAL	Dallas, TX	MOD	Modesto, CA	STL	St Louis, MO
DEN	Denver, CO	MSN	Madison, WI	TOL	Toledo, OH
DFW	Fort Worth, TX	MSP	Minneapolis, MN	TPA	Tampa, FL
DTT	Detroit, MI	MSY	New Orleans, LA	TUL	Tulsa, OK
ELP	El Paso, TX	NHE	North Hempstead, NY	TUS	Tucson, AZ
EWR	Newark, NJ	NYC	New York, NY	VIB	Virginia Beach, VA
FAT	Fresno, CA	OAK	Oakland, CA	WAS	Washington, DC
FWA	Fort Wayne, IN				

Table 1

Quantity	Mean	Standard Dev.
Number of users (daily)	9,903,993	3,447,776
Number of users who updated (daily)	2,042,996	775,162
Number of friendships (daily)	52,787,239	25,118,462

Table 2: For each day in the period of observation (a set of 1180 days from January 2009 to March 2012) all Facebook users that are English-speakers and geolocated within the 100 most populous US cities are included. Assuming that each user posts either one or zero status updates on a day, the average number of status updates per user per day is $\alpha = 0.206$.

Summary of Emotion and Meteorological Variables				
	Mean	Standard Deviation	Minimum	Maximum
Positive rate	0.407	0.0445	0.116	0.614
Negative rate	0.213	0.0329	0.0388	0.440
Weather posts	0.0653	0.0347	0	0.527
Rainfall indicator	0.257	0.437	0	1

Table 3: The summary statistics for each emotional and meteorological variable are computed considering one observation for each city-day pair.

Summary of Rainfall in each City											
City Code	Num Days	Rainy Days	City Code	Num Days	Rainy Days	City Code	Num Days	Rainy Days	City Code	Num Days	Rainy Days
ABQ	120	1180	CVG	379	1180	LEX	236	1180	PIT	378	1180
ANA	424	1180	DAL	257	1180	LGB	404	1180	PLA	386	1180
ANC	278	1180	DEN	217	1180	LNK	274	1180	RAL	94	1180
ATL	139	1180	DFW	350	1180	LRD	358	1180	RDU	241	1180
AUR	236	1180	DTT	566	1180	MCI	280	1180	RIV	84	1180
AUS	243	1180	ELP	132	1180	MEM	131	1180	RNO	377	1180
AWO	94	1180	EWR	217	1180	MES	303	1180	ROC	113	1180
BFL	438	1180	FAT	137	1180	MGM	102	1180	SAN	357	1180
BHM	303	1180	FWA	148	1180	MIA	446	1180	SAT	384	1180
BNA	207	1180	GAR	341	1180	MKE	558	1180	SBD	402	1180
BOI	344	1180	GEU	132	1180	MOD	132	1180	SCK	116	1180
BOS	460	1180	GKY	389	1180	MSN	408	1180	SDL	213	1180
BTR	420	1180	GSP	347	1180	MSP	257	1180	SEA	519	1180
BUF	226	1180	HIA	214	1180	MSY	450	1180	SFO	461	1180
BWI	207	1180	HND	519	1180	NHE	341	1180	SJC	370	1180
CAK	372	1180	HNL	84	1180	NYC	523	1180	SMF	443	1180
CHD	342	1180	HOU	373	1180	OAK	382	1180	SNA	183	1180
CHI	243	1180	HTS	323	1180	OKC	243	1180	SNP	177	1180
CHU	407	1180	ICT	153	1180	OMA	444	1180	STL	536	1180
CLE	131	1180	IND	256	1180	ORF	299	1180	TOL	196	1180
CLT	397	1180	JAX	267	1180	ORL	296	1180	TPA	305	1180
CMH	390	1180	JCY	414	1180	PDX	369	1180	TUL	334	1180
COS	388	1180	LAS	283	1180	PHL	92	1180	TUS	400	1180
CPK	365	1180	LAX	362	1180	PHX	311	1180	VIB	320	1180
CRP	562	1180	LBB	134	1180	PIE	154	1180	WAS	388	1180

Table 4

Emotion measure: positive rate (non rainy days)<br>Instrument: binary indicator of rainfall						
FIRST STAGE<br>Friends' emotion $\bar{Y}^{(p)}$	Coefficient	Standard<br>Error	t	$P > \|t\|$	95% Confidence Interval<br>Low	High
Friends' rainfall $\bar{X}$	-0.0119	0.00207	-5.75	0.000	-0.0160	-.00781
SECOND STAGE<br>Users' emotion $\bar{y}^{(p)}$	Coefficient	Standard<br>Error	t	$P > \|t\|$	95% Confidence Interval<br>Low	High
Friends' emotion $\bar{Y}^{(p)}$	1.752	0.122	14.39	0.000	1.514	1.991

Table 5: Observations such that $\bar{x}_{gt} = 0$ are considered (87,700 total observations). The Kleibergen-Paap rk LM statistic is 25.507 ($p = 0.0000$) suggesting the regression is not underidentified.[3] The Cragg-Donald Wald F statistic is 324.053, which exceeds the critical thresholds suggested by Stock and Yogo [5] to ensure the instruments are not weak. All statistics are robust to heteroskedasticity, autocorrelation, and clustering.

Emotion measure: negative rate (non rainy days)<br>Instrument: binary indicator of rainfall						
FIRST STAGE<br>Friends' emotion $\bar{Y}^{(n)}$	Coefficient	Standard<br>Error	t	$P > \|t\|$	95% Confidence Interval<br>Low	High
Friends' rainfall $\bar{X}$	0.0116	0.00195	5.97	0.000	0.00776	0.0155
SECOND STAGE<br>Users' emotion $\bar{y}^{(n)}$	Coefficient	Standard<br>Error	t	$P > \|t\|$	95% Confidence Interval<br>Low	High
Friends' emotion $\bar{Y}^{(n)}$	1.288	0.0486	26.53	0.000	1.193	1.383

Table 6: Observations such that $\bar{x}_{gt} = 0$ are considered (87,700 total observations). The Kleibergen-Paap rk LM statistic is 24.598 ($p = 0.0000$) suggesting the regression is not underidentified.[3] The Cragg-Donald Wald F statistic is 505.398, which exceeds the critical thresholds suggested by Stock and Yogo [5] to ensure the instruments are not weak. All statistics are robust to heteroskedasticity, autocorrelation, and clustering.

How friends' positive rate affects users' negative rate (non rainy days)<br>Instrument: binary indicator of rainfall						
FIRST STAGE<br>Friends' emotion $\bar{Y}^{(p)}$	Coefficient	Standard<br>Error	t	$P > \|t\|$	95% Confidence Interval<br>Low	High
Friends' rainfall $\bar{X}$	-0.0119	0.00207	-5.75	0.000	-0.0160	-0.00781
SECOND STAGE<br>User' emotion $\bar{y}^{(n)}$	Coefficient	Standard<br>Error	t	$P > \|t\|$	95% Confidence Interval<br>Low	High
Friends' emotion $\bar{Y}^{(p)}$	-1.255	0.227	-5.52	0.000	-1.701	-0.809

Table 7: Observations such that $\bar{x}_{gt} = 0$ are considered (87,700 total observations). The Kleibergen-Paap rk LM statistic is 25.507 ($p = 0.0000$) suggesting the regression is not underidentified.[3] TheCragg-Donald Wald F statistic is 324.053, which exceeds the critical thresholds suggested by Stock and Yogo [5] to ensure the instruments are not weak. All statistics are robust to heteroskedasticity, autocorrelation, and clustering.

How friends' negative rate affects users' positive rate (non rainy days)<br>Instrument: binary indicator of rainfall						
FIRST STAGE<br>Friends' emotion $\bar{Y}^{(n)}$	Coefficient	Standard<br>Error	t	$P > \|t\|$	95% Confidence Interval<br>Low	High
Friends' rainfall $\bar{X}$	0.0116	0.00195	5.97	0.000	0.00776	0.0155
SECOND STAGE<br>User' emotion $\bar{y}^{(p)}$	Coefficient	Standard<br>Error	t	$P > \|t\|$	95% Confidence Interval<br>Low	High
Friends' emotion $\bar{Y}^{(n)}$	-1.798	0.271	-6.62	0.000	-2.330	-1.266

Table 8: Observations such that $\bar{x}_{gt} = 0$ are considered (87,700 total observations). The Kleibergen-Paap rk LM statistic is 24.598 ($p = 0.0000$) suggesting the regression is not underidentified.[3] TheCragg-Donald Wald F statistic is 505.398, which exceeds the critical thresholds suggested by Stock and Yogo [5] to ensure the instruments are not weak. All statistics are robust to heteroskedasticity, autocorrelation, and clustering.

Emotion measure: positive rate (rainy days)<br>Instrument: binary indicator of rainfall						
FIRST STAGE<br>Friends' emotion $\bar{Y}^{(p)}$	Coefficient	Standard<br>Error	t	$P > \|t\|$	95% Confidence Interval<br>Low	 High
Friends' rainfall $\bar{X}$	-0.00985	0.00268	-3.68	0.000	-0.0152	-0.00454
SECOND STAGE<br>Users' emotion $\bar{y}^{(p)}$	Coefficient	Standard<br>Error	t	$P > \|t\|$	95% Confidence Interval<br>Low	 High
Friends' emotion $\bar{Y}^{(p)}$	1.794	0.233	7.70	0.000	1.338	2.251

Table 9: Observations such that $\bar{x}_{gt} = 1$ are considered (30,300 total observations). The Kleibergen-Paap rk LM statistic is 13.531 ($p = 0.0002$) suggesting the regression is not underidentified.[3] The Cragg-Donald Wald F statistic is 78.189, which exceeds the critical thresholds suggested by Stock and Yogo [5] to ensure the instruments are not weak. All statistics are robust to heteroskedasticity, autocorrelation, and clustering.

Emotion measure: negative rate (rainy days)<br>Instrument: binary indicator of rainfall						
FIRST STAGE<br>Friends' emotion $\bar{Y}^{(n)}$	Coefficient	Standard<br>Error	t	$P > \|t\|$	95% Confidence Interval<br>Low	 High
Friends' rainfall $\bar{X}$	0.00973	0.00281	3.47	0.001	0.00416	0.0153
SECOND STAGE<br>Users' emotion $\bar{y}^{(n)}$	Coefficient	Standard<br>Error	t	$P > \|t\|$	95% Confidence Interval<br>Low	 High
Friends' emotion $\bar{Y}^{(n)}$	1.473	0.134	10.97	0.000	1.210	1.736

Table 10: Observations such that $\bar{x}_{gt} = 1$ are considered (30,300 total observations). The Kleibergen-Paap rk LM statistic is 11.333 ($p = 0.0008$) suggesting the regression is not underidentified.[3] The Cragg-Donald Wald F statistic is 102.297, which exceeds the critical thresholds suggested by Stock and Yogo [5] to ensure the instruments are not weak. All statistics are robust to heteroskedasticity, autocorrelation, and clustering.

How friends' positive rate affects users' negative rate (rainy days)<br>Instrument: binary indicator of rainfall						
FIRST STAGE<br>Friends' emotion $\bar{Y}^{(p)}$	Coefficient	Standard<br>Error	t	$P > \|t\|$	95% Confidence Interval<br>Low	 High
Friends' rainfall $\bar{X}$	-0.00985	0.00268	-3.68	0.000	-0.0152	-0.00454
SECOND STAGE<br>User' emotion $\bar{y}^{(n)}$	Coefficient	Standard<br>Error	t	$P > \|t\|$	95% Confidence Interval<br>Low	 High
Friends' emotion $\bar{Y}^{(p)}$	-1.456	0.475	-3.06	0.002	-2.387	-0.524

Table 11: Observations such that $\bar{x}_{gt} = 1$ are considered (30,300 total observations). The Kleibergen-Paap rk LM statistic is 13.531 ($p = 0.0002$) suggesting the regression is not underidentified.[3] TheCragg-Donald Wald F statistic is 78.189, which exceeds the critical thresholds suggested by Stock and Yogo [5] to ensure the instruments are not weak. All statistics are robust to heteroskedasticity, autocorrelation, and clustering.

How friends' negative rate affects users' positive rate (rainy days)<br>Instrument: binary indicator of rainfall						
FIRST STAGE<br>Friends' emotion $\bar{Y}^{(n)}$	Coefficient	Standard<br>Error	t	$P > \|t\|$	95% Confidence Interval<br>Low	 High
Friends' rainfall $\bar{X}$	0.00973	0.00281	3.47	0.001	0.00416	0.0153
SECOND STAGE<br>User' emotion $\bar{y}^{(p)}$	Coefficient	Standard<br>Error	t	$P > \|t\|$	95% Confidence Interval<br>Low	 High
Friends' emotion $\bar{Y}^{(n)}$	-1.816	0.550	-3.30	0.001	-2.895	-0.738

Table 12: Observations such that $\bar{x}_{gt} = 1$ are considered (30,300 total observations). The Kleibergen-Paap rk LM statistic is 11.333 ($p = 0.0008$) suggesting the regression is not underidentified.[3] TheCragg-Donald Wald F statistic is 102.297, which exceeds the critical thresholds suggested by Stock and Yogo [5] to ensure the instruments are not weak. All statistics are robust to heteroskedasticity, autocorrelation, and clustering.

Model in Equation (11) (non rainy days)<br>Emotion measure: positive rate - Instrument: binary indicator of rainfall						
FIRST STAGE<br>Friends' emotion $\bar{Y}^{(p)}_{t+30}$	Coefficient	Standard<br>Error	t	$P > \|t\|$	95% Confidence Interval<br>Low	 High
Friends' rainfall $\bar{X}_{t+30}$	-0.0118	0.00191	-6.19	0.000	-0.0156	-0.00804
SECOND STAGE<br>Users' emotion $\bar{y}^{(p)}$	Coefficient	Standard<br>Error	t	$P > \|t\|$	95% Confidence Interval<br>Low	 High
Friends' emotion $\bar{Y}^{(p)}_{t+30}$	-0.112	0.177	-0.63	0.526	-0.459	0.235

Table 13: Observations such that $\bar{x}_{gt} = 0$ and $\bar{x}_{g,t+30} = 0$ are considered (67,493 total observations). The Kleibergen-Paap rk LM statistic is 28.711 ($p = 0.0000$) suggesting the regression is not underidentified.[3] The Cragg-Donald Wald F statistic is 265.910, which exceeds the critical thresholds suggested by Stock and Yogo [5] to ensure the instruments are not weak. All statistics are robust to heteroskedasticity, autocorrelation, and clustering.

Model in Equation (11) (non rainy days)<br>Emotion measure: negative rate - Instrument: binary indicator of rainfall						
FIRST STAGE<br>Friends' emotion $\bar{Y}^{(n)}_{t+30}$	Coefficient	Standard<br>Error	t	$P > \|t\|$	95% Confidence Interval<br>Low	 High
Friends' rainfall $\bar{X}_{t+30}$	0.0120	0.00188	6.42	0.000	0.00832	0.0158
SECOND STAGE<br>Users' emotion $\bar{y}^{(n)}$	Coefficient	Standard<br>Error	t	$P > \|t\|$	95% Confidence Interval<br>Low	 High
Friends' emotion $\bar{Y}^{(n)}_{t+30}$	-0.185	0.126	-1.46	0.143	-0.432	0.0627

Table 14: Observations such that $\bar{x}_{gt} = 0$ and $\bar{x}_{g,t+30} = 0$ are considered (67,493 total observations). The Kleibergen-Paap rk LM statistic is 26.552 ($p = 0.0000$) suggesting the regression is not underidentified.[3] The Cragg-Donald Wald F statistic is 458.685, which exceeds the critical thresholds suggested by Stock and Yogo [5] to ensure the instruments are not weak. All statistics are robust to heteroskedasticity, autocorrelation, and clustering.

Model in Equation (11) (non rainy days)<br>Friends' positive rate to users' negative rate - Instrument: binary indicator of rainfall						
FIRST STAGE<br>Friends' emotion $\bar{Y}^{(p)}_{t+30}$	Coefficient	Standard<br>Error	t	$P > \|t\|$	95% Confidence Interval<br>Low	 High
Friends' rainfall $\bar{X}_{t+30}$	-0.0118	0.00191	-6.19	0.000	-0.0156	-0.00804
SECOND STAGE<br>User' emotion $\bar{y}^{(n)}$	Coefficient	Standard<br>Error	t	$P > \|t\|$	95% Confidence Interval<br>Low	 High
Friends' emotion $\bar{Y}^{(p)}_{t+30}$	0.188	0.121	1.55	0.120	-0.0493	0.425

Table 15: Observations such that $\bar{x}_{gt} = 0$ and $\bar{x}_{g,t+30} = 0$ are considered (67,493 total observations). The Kleibergen-Paap rk LM statistic is 28.711 ($p = 0.0000$) suggesting the regression is not underidentified.[3] TheCragg-Donald Wald F statistic is 265.910, which exceeds the critical thresholds suggested by Stock and Yogo [5] to ensure the instruments are not weak. All statistics are robust to heteroskedasticity, autocorrelation, and clustering.

Model in Equation (11) (non rainy days)<br>Friends' negative rate to users' positive rate - Instrument: binary indicator of rainfall						
FIRST STAGE<br>Friends' emotion $\bar{Y}^{(n)}_{t+30}$	Coefficient	Standard<br>Error	t	$P > \|t\|$	95% Confidence Interval<br>Low	 High
Friends' rainfall $\bar{X}_{t+30}$	0.0120	0.00188	6.42	0.000	0.00832	0.0158
SECOND STAGE<br>User' emotion $\bar{y}^{(p)}$	Coefficient	Standard<br>Error	t	$P > \|t\|$	95% Confidence Interval<br>Low	 High
Friends' emotion $\bar{Y}^{(n)}_{t+30}$	0.110	0.172	0.64	0.521	-0.226	0.447

Table 16: Observations such that $\bar{x}_{gt} = 0$ and $\bar{x}_{g,t+30} = 0$ are considered (67,493 total observations). The Kleibergen-Paap rk LM statistic is 26.552 ($p = 0.0000$) suggesting the regression is not underidentified.[3] TheCragg-Donald Wald F statistic is 458.685, which exceeds the critical thresholds suggested by Stock and Yogo [5] to ensure the instruments are not weak. All statistics are robust to heteroskedasticity, autocorrelation, and clustering.

aerovane air airstream altocumulus altostratus anemometer anemometers anticyclone anticyclones arctic arid aridity atmosphere atmospheric autumn autumnal balmy baroclinic barometer barometers barometric blizzard blizzards blustering blustery blustery breeze breezes breezy brisk calm celsius chill chilled chillier chilliest chilly chinook cirrocumulus cirrostratus cirrus climate climates cloud cloudburst cloudbursts cloudier cloudiest clouds cloudy cold colder coldest condensation contrail contrails cool cooled cooling cools cumulonimbus cumulus cyclone cyclones damp damp damper damper dampest dampest degree degrees deluge dew dews dewy doppler downburst downbursts downdraft downdrafts downpour downpours dried drier dries driest drizzle drizzled drizzles drizzly drought droughts dry dryline fall farenheit flood flooded flooding floods flurries flurry fog fogbow fogbows fogged fogging foggy fogs forecast forecasted forecasting forecasts freeze freezes freezing frigid frost frostier frostiest frosts frosty froze frozen gale gales galoshes gust gusting gusts gusty haboob haboobs hail hailed hailing hails haze hazes hazy heat heated heating heats hoarfrost hot hotter hottest humid humidity hurricane hurricanes ice iced ices icing icy inclement landspout landspouts lightning lightnings macroburst macrobursts maelstrom mercury meteorologic meteorologist meteorologists meteorology microburst microbursts microclimate microclimates millibar millibars mist misted mists misty moist moisture monsoon monsoons mugginess muggy nexrad nippy NOAA nor'easter nor'easters noreaster noreasters overcast ozone parched parching pollen precipitate precipitated precipitates precipitating precipitation psychrometer radar rain rainboots rainbow rainbows raincoat raincoats rained rainfall rainier rainiest raining rains rainy sandstorm sandstorms scorcher scorching searing shower showering showers skiff sleet slicker slickers slush slushy smog smoggier smoggiest smoggy snow snowed snowier snowiest snowing snowmageddon snowpocalypse snows snowy spring sprinkle sprinkles sprinkling squall squalls squally storm stormed stormier stormiest storming storms stormy stratocumulus stratus subtropical summer summery sun sunnier sunniest sunny temperate temperature tempest thaw thawed thawing thaws thermometer thunder thundered thundering thunders thunderstorm thunderstorms tornadic tornado tornadoes tropical troposphere tsunami turbulent twister twisters typhoon typhoons umbrella umbrellas vane warm warmed warming warms warmth waterspout waterspouts weather wet wetter wettest wind windchill windchills windier windiest windspeed windy winter wintery wintry

Table 17: Terms used to identify status updates on the topic of weather.

Model in Equation (12) (non rainy days)						
Emotion measure: positive rate - Instrument: binary indicator of rainfall						
FIRST STAGE		Standard			95% Confidence Interval	
Friends' emotion $\bar{Y}^{(p)}$	Coefficient	Error	t	$P > \|t\|$	Low	High
Friends' rainfall $\bar{X}$	-0.00888	0.00199	-4.46	0.000	-0.0128	-0.00492
Users' weather rate $\bar{w}$	-0.0186	0.00322	-5.78	0.000	-0.0250	-0.0122
SECOND STAGE		Standard			95% Confidence Interval	
Users' emotion $\bar{y}^{(p)}$	Coefficient	Error	t	$P > \|t\|$	Low	High
Friends' emotion $\bar{Y}^{(p)}$	1.205	0.0974	12.37	0.000	1.0140	1.396
Users' weather rate $\bar{w}$	-0.0399	0.00301	-13.25	0.000	-0.0458	-0.0340

Table 18: Observations such that $\bar{x}_{gt} = 0$ are considered (87,700 total observations). The Kleibergen-Paap rk LM statistic is 17.750 ($p = 0.0000$) suggesting the regression is not underidentified.[3] The Cragg-Donald Wald F statistic is 169.315, which exceeds the critical thresholds suggested by Stock and Yogo [5] to ensure the instruments are not weak. All statistics are robust to heteroskedasticity, autocorrelation, and clustering.

Model in Equation (12) (non rainy days)<br>Emotion measure: negative rate - Instrument: binary indicator of rainfall						
FIRST STAGE<br>Friends' emotion $\bar{Y}^{(n)}$	Coefficient	Standard<br>Error	t	$P > \|t\|$	95% Confidence Interval<br>Low	 High
Friends' rainfall $\bar{X}$	0.00749	0.00175	4.29	0.000	0.00403	0.0110
Users' weather rate $\bar{w}$	0.0252	0.00496	5.09	0.000	0.0154	0.0351
SECOND STAGE<br>Users' emotion $\bar{y}^{(n)}$	Coefficient	Standard<br>Error	t	$P > \|t\|$	95% Confidence Interval<br>Low	 High
Friends' emotion $\bar{Y}^{(n)}$	1.509	0.0986	15.30	0.000	1.315	1.702
Users' weather rate $\bar{w}$	-0.0157	0.00305	-5.14	0.000	-0.0217	-0.00971

Table 19: Observations such that $\bar{x}_{gt} = 0$ are considered (87,700 total observations). The Kleibergen-Paap rk LM statistic is 15.799 ($p = 0.0001$) suggesting the regression is not underidentified.[3] The Cragg-Donald Wald F statistic is 199.681, which exceeds the critical thresholds suggested by Stock and Yogo [5] to ensure the instruments are not weak. All statistics are robust to heteroskedasticity, autocorrelation, and clustering.

Model in Equation (12) (non rainy days)<br>Friends' positive rate to users' negative rate - Instrument: binary indicator of rainfall						
FIRST STAGE<br>Friends' emotion $\bar{Y}^{(p)}$	Coefficient	Standard<br>Error	t	$P > \|t\|$	95% Confidence Interval<br>Low	 High
Friends' rainfall $\bar{X}$	-0.00888	0.00199	-4.46	0.000	-0.0128	-0.00492
Users' weather rate $\bar{w}$	-0.0186	0.00322	-5.78	0.000	-0.0250	-0.0122
SECOND STAGE<br>User' emotion $\bar{y}^{(n)}$	Coefficient	Standard<br>Error	t	$P > \|t\|$	95% Confidence Interval<br>Low	 High
Friends' emotion $\bar{Y}^{(p)}$	-1.274	0.302	-4.22	0.000	-1.866	-0.681
Users' weather rate $\bar{w}$	-0.00134	0.0113	-0.12	0.905	-0.0234	0.0207

Table 20: Observations such that $\bar{x}_{gt} = 0$ are considered (87,700 total observations). The Kleibergen-Paap rk LM statistic is 17.750 ($p = 0.0000$) suggesting the regression is not underidentified.[3] TheCragg-Donald Wald F statistic is 169.315, which exceeds the critical thresholds suggested by Stock and Yogo [5] to ensure the instruments are not weak. All statistics are robust to heteroskedasticity, autocorrelation, and clustering.

Model in Equation (12) (non rainy days)<br>Friends' negative rate to users' positive rate - Instrument: binary indicator of rainfall						
FIRST STAGE<br>Friends' emotion $\bar{Y}^{(n)}$	Coefficient	Standard<br>Error	t	$P > \|t\|$	95% Confidence Interval<br>Low	 High
Friends' rainfall $\bar{X}$	0.00749	0.00175	4.29	0.000	0.00403	0.0110
Users' weather rate $\bar{w}$	0.0252	0.00496	5.09	0.000	0.0154	0.0351
SECOND STAGE<br>User' emotion $\bar{y}^{(p)}$	Coefficient	Standard<br>Error	t	$P > \|t\|$	95% Confidence Interval<br>Low	 High
Friends' emotion $\bar{Y}^{(n)}$	-1.427	0.368	-3.88	0.000	-2.149	-0.706
Users' weather rate $\bar{w}$	-0.0264	0.0150	-1.76	0.078	-0.0557	0.00300

Table 21: Observations such that $\bar{x}_{gt} = 0$ are considered (87,700 total observations). The Kleibergen-Paap rk LM statistic is 15.799 ($p = 0.0001$) suggesting the regression is not underidentified.[3] TheCragg-Donald Wald F statistic is 199.681, which exceeds the critical thresholds suggested by Stock and Yogo [5] to ensure the instruments are not weak. All statistics are robust to heteroskedasticity, autocorrelation, and clustering.

Average user emotional contagion effect – estimates and 95% CI				
User's emotion	Friend's emotion	$\bar{D}_t$	Lo95%	Hi95%
Positive rate	Positive rate	1.752	1.514	1.991
Negative rate	Negative rate	1.288	1.193	1.383
Positive rate	Negative rate	-1.255	-1.701	-0.809
Negative rate	Positive rate	-1.798	-2.330	-1.266

Table 22

Indirect and direct effect of rain in a city – estimates and 95% CI (Number of negative posts)							
	Population	Indirect effect			Direct effect		
City Code	(US Census 2010)	$\bar{I}_g$	Lo95%	Hi95%	$\bar{D}_g$	Lo95%	Hi95%
NYC	8175133	712.14	626.23	806.32	1550.42	1023.81	2071.68
LAX	3792621	666.96	586.5	755.16	1136.34	750.37	1518.38
CHI	2695598	573.32	504.16	649.14	1637.96	1081.62	2188.65
WAS	601723	494.37	434.73	559.75	878.38	580.04	1173.7
ATL	420003	477.59	419.98	540.75	992.6	655.46	1326.32
DAL	1197816	445.36	391.63	504.25	714.63	471.9	954.89
HOU	2100263	352.2	309.71	398.77	881.18	581.88	1177.44
SAN	1307402	299.02	262.95	338.56	536.04	353.97	716.26
AUS	790390	283.22	249.05	320.67	443.31	292.74	592.35
SFO	805235	278.62	245.01	315.47	419.23	276.84	560.18
ORL	238300	262.76	231.06	297.51	530.92	350.59	709.42
PHX	1445632	252.38	221.94	285.76	432.45	285.57	577.85
PHL	1526006	250.07	219.9	283.14	831.29	548.94	1110.77
BOS	617594	226.16	198.87	256.07	678.99	448.37	907.27
LAS	583756	215.53	189.53	244.03	367.55	242.71	491.12
TPA	335709	203.99	179.38	230.96	407.76	269.26	544.85
CLT	731424	202.33	177.92	229.09	441.28	291.4	589.65
BWI	620961	201.65	177.32	228.32	510.88	337.36	682.64
SEA	608660	199.65	175.57	226.05	450.34	297.38	601.75
MSP	382578	189.01	166.21	214	487.64	322.01	651.58
MIA	399457	184.1	161.89	208.44	511.4	337.7	683.34
BNA	601222	181.93	159.98	205.99	340.53	224.87	455.02
SAT	1327407	180.27	158.52	204.11	402.74	265.95	538.14
DEN	600158	174.49	153.44	197.57	342.77	226.35	458.01
DTT	713777	169.29	148.86	191.67	722.21	476.91	965.02
CMH	787033	165.41	145.45	187.28	385.78	254.75	515.48
VIB	437994	164.3	144.48	186.03	261.33	172.57	349.19
RAL	403892	155.61	136.84	176.19	331.11	218.64	442.43
PDX	583776	151.09	132.86	171.07	368.13	243.1	491.9
IND	820445	145.44	127.89	164.67	426.14	281.4	569.42
STL	319294	140.84	123.85	159.47	428.03	282.65	571.94
PIT	305704	138.34	121.65	156.63	423.86	279.89	566.36
JAX	821784	128.47	112.97	145.46	313.3	206.88	418.63
CVG	296943	125.75	110.58	142.38	349.55	230.82	467.07
MKE	594833	123.44	108.55	139.76	356.36	235.32	476.17
CLE	396815	121.64	106.97	137.73	290.14	191.59	387.69
MCI	459787	121.57	106.9	137.64	361.38	238.64	482.88
MSY	343829	119.4	104.99	135.18	217.86	143.86	291.1
DFW	741206	114.47	100.66	129.6	143.96	95.06	192.36
SMF	466488	109.26	96.08	123.7	259.26	171.2	346.42
MEM	646889	101.05	88.86	114.41	281.99	186.21	376.8
COS	416427	85.02	74.76	96.26	164.61	108.7	219.95
TUS	520116	85	74.74	96.24	183.67	121.29	245.42
OKC	579999	82.09	72.19	92.95	197.24	130.25	263.55
BHM	212237	80.15	70.48	90.74	206.74	136.52	276.25
ROC	210565	79.8	70.18	90.36	231	152.54	308.66
GSP	269666	79.29	69.72	89.77	148.43	98.02	198.34
BUF	261310	78.59	69.11	88.99	240.16	158.59	320.91
OMA	408958	77.38	68.04	87.61	198.7	131.21	265.51
HNL	337256	76.94	67.66	87.11	146.49	96.73	195.74

Table 23

Indirect and direct effect of rain in a city – estimates and 95% CI (Number of negative posts)							
	Population	Indirect effect			Direct effect		
City Code	(US Census 2010)	$\bar{I}_g$	Lo95%	Hi95%	$\bar{D}_g$	Lo95%	Hi95%
MSN	233209	75.29	66.21	85.25	157.48	103.99	210.42
TUL	391906	73.27	64.43	82.96	182.92	120.79	244.42
LGB	462257	70.16	61.7	79.44	82.38	54.4	110.08
BTR	229493	69.06	60.73	78.19	144.26	95.26	192.76
GKY	365438	68.67	60.38	77.75	77.07	50.89	102.98
ORF	242803	66.44	58.43	75.23	69.23	45.72	92.51
SDL	217385	64.1	56.37	72.58	56.15	37.08	75.03
SJC	945942	63.62	55.95	72.04	124.93	82.5	166.93
LEX	295803	62.26	54.75	70.49	166.71	110.08	222.76
MES	439041	58.33	51.29	66.04	70.82	46.76	94.63
SNP	285068	58.13	51.12	65.82	56.62	37.39	75.65
AWO	207627	58.11	51.1	65.79	48.02	31.71	64.16
CAK	199110	54.97	48.34	62.24	127.26	84.03	170.04
ICT	382368	53.72	47.24	60.82	172.3	113.78	230.23
OAK	390724	52.66	46.31	59.62	63	41.6	84.17
PLA	259841	52.58	46.23	59.53	51.25	33.84	68.47
TOL	287208	52.5	46.17	59.44	139.47	92.1	186.36
ABQ	545852	51.48	45.27	58.29	124.55	82.25	166.42
LNK	258379	48.66	42.79	55.09	113.83	75.17	152.11
LBB	229573	45.6	40.1	51.63	83.81	55.34	111.98
CPK	222209	44.53	39.16	50.42	48.25	31.86	64.47
CHD	236123	42.98	37.8	48.67	44.24	29.21	59.11
FAT	494665	40.7	35.79	46.08	114.99	75.94	153.66
JCY	247597	40.27	35.41	45.6	79.44	52.46	106.15
FWA	253691	39.61	34.83	44.85	112.57	74.33	150.42
ELP	649121	38.59	33.94	43.69	89.57	59.15	119.68
MGM	205764	38.23	33.62	43.29	83.39	55.07	111.43
CRP	305215	36.17	31.81	40.95	72.06	47.58	96.28
BOI	205671	34.14	30.02	38.66	98.93	65.33	132.19
ANC	291826	33.83	29.75	38.3	82.87	54.72	110.73
PIE	244769	33.8	29.72	38.27	36.59	24.16	48.89
HND	257729	33.54	29.49	37.97	32.71	21.6	43.71
RNO	225221	30.89	27.16	34.97	67.57	44.62	90.29
BFL	347483	27.67	24.33	31.33	84.18	55.59	112.49
RIV	303871	25.28	22.23	28.62	48.15	31.79	64.33
GAR	226876	23.98	21.08	27.15	26.58	17.55	35.52
SCK	291707	19	16.71	21.51	40.11	26.49	53.6
HTS	189992	18.27	16.07	20.69	49	32.36	65.48
ANA	336265	18.18	15.99	20.59	30.24	19.97	40.41
CHU	243916	17.9	15.74	20.27	18.21	12.03	24.34
MOD	201165	15.42	13.56	17.46	37.16	24.54	49.65
GEU	226721	14.99	13.18	16.97	13.86	9.15	18.52
EWR	277140	13.44	11.81	15.21	27.85	18.39	37.21
HIA	224669	11.27	9.91	12.76	12.02	7.94	16.06
LRD	236091	9.46	8.31	10.71	27.13	17.92	36.25
SNA	324528	8.65	7.61	9.8	17.44	11.52	23.31
SBD	209924	7.51	6.6	8.5	15.98	10.55	21.36
NHE	226322	5.35	4.71	6.06	6.01	3.97	8.03
RDU	228330	1.22	1.07	1.38	28.31	18.69	37.82
AUR	325078	0.31	0.27	0.35	3.87	2.55	5.17

Table 24

Bibliography

[1] JW Pennebaker, CK Chung, M Ireland, A Gonzales, RJ Booth, The development and psychological properties of LIWC2007. http://www.liwc.net.

[2] JM Wooldridge (2001), Econometric analysis of cross section and panel data. MIT press.

[3] Kleibergen, F, and Paap, R (2006). Generalized reduced rank tests using the singular- value decomposition. Journal of Econometrics 127: 97126.

[4] Waxman, B. M. (1988). Routing of multipoint connections. IEEE Journal on Selected Areas in Communications 6(9), 1617-1622.

[5] Stock, J. H., and M. Yogo (2005), Testing for weak instruments in linear IV regression. In Identification and Inference for Econometric Models: Essays in Honor of Thomas Rothenberg, ed. D. W. K. Andrews and J. H. Stock, 80108. Cambridge University Press.

[6] CF Baum, ME Schaffer, S Stillman (2007), Enhanced routines for instrumental variables/GMM estimation and testing, Stata Journal 7(4):465–506.
